# Supplementary material for: IRE1a-Induced FilaminA Phosphorylation Enhances Migration of Mesenchymal Stem Cells Derived from Multiple Myeloma Patients
Source: Cells. 2023 Jul 26;12(15):1935. doi: 10.3390/cells12151935 (PMC10417526; doi:10.3390/cells12151935)
Supplement: Supplementary file 1 [file cells-12-01935-s001.zip › cells-2456653-supplementary.pdf]

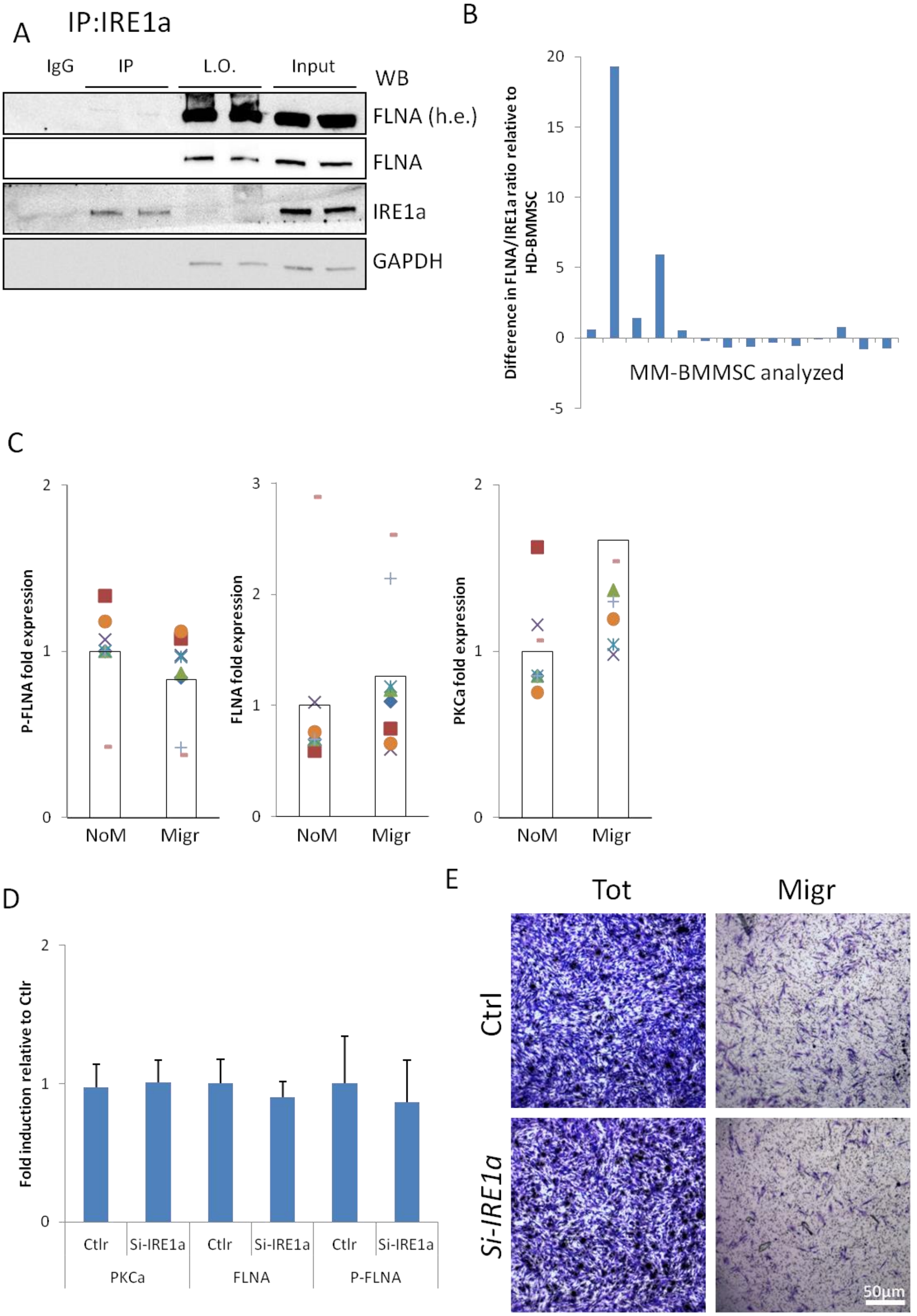

Supplementary Figure S1: A) Immunoprecipitation (IP) for IRE1a and following wb in BMMSC samples. Negative control (IgG), left over (L.O.) and input are also showed. H.e.= high exposure. B) Ratio FLNA/IRE1a after immunoprecipitation in MM-BMMSC samples in non migrating condition. Value are normalized to HD-BMMSc level set as 1. C) Relative expression of P-FLNA (S2152), FLNA and PKCa in non migrating and

migrating BMMSC. n=8. Two tail t-test. D) Analysis of protein level in BMMSC after downregulation of IRE1a with Si-ERN1. Results of 3 experiments; both Ctrl and *Si-ERN1* 5<n<7. E) Representative images of total (left) and migrated (right) BMMSC in Ctrl and *Si-ERN1* condition; Crystal violet staining. Scale bar 50µm

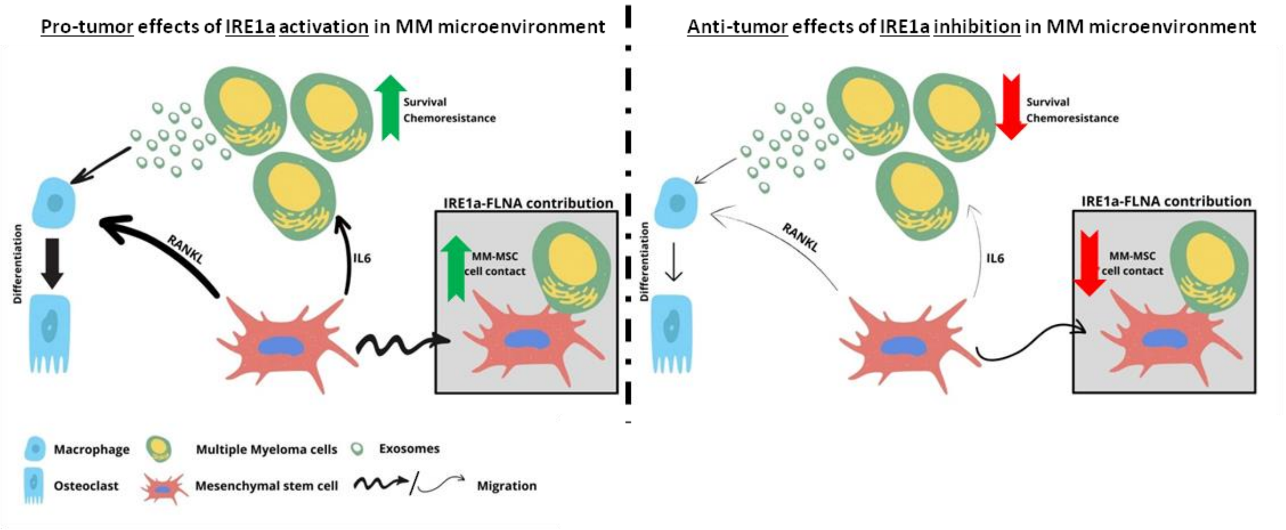

Supplementary Figure S2. Update about IRE1a contribution in MM microenvironment

In MM IRE1a regulates pro-tumor microenvironment (left panel) increasing directly tumor cell survival and chemoresistance (Harnoss at al., 2019; Mimura et al., 2012; Papandreou et al., 2011; Yamashita et al., 2020). In parallel, IRE1a-XBP1s activation, by MM-derived vesicles, induces osteoclastogenic macrophage differentiation worsening the structure of bone tissue (Raimondi et al., 2020). Moreover MM-BMMSC possess an enhanced secretion of RANKL and IL6 that is dependent by IRE1a-XBP1s (Xu et al., 2012b). We found that IRE1a-FLNA axis contributes to migratory capability of BMMSC and we hypothesize (gray square) that this process, in MM contest, produces an higher probability of MM/BMMSC direct contact and cell adhesion-mediated drug resistance (CAM-DR) (Meads et al., 2009). We are confident that IRE1a can be a putative common target to counteract MM progression in different aspects, as highlighted by thinner arrows in the right panel. Inhibition of IRE1a activity has a direct effect on viability of MM cells, it reduces chemoresistance (Harnoss at al., 2019; Mimura et al., 2012; Papandreou et al., 2011; Yamashita et al., 2020) and osteoclasts differentiation and it interferes with pro-tumor behavior of BMMSC. In addiction inhibition of migration, through IRE1a-FLNA axis inhibition, could impact on MM and BMMSC interaction and on manifestation of cell-adhesion-mediated drug resistance.
